# Primary oocytes with cellular senescence features are involved in ovarian aging in mice

**DOI:** 10.1038/s41598-024-64441-6

**Published:** 2024-06-13

**Authors:** Hao Yan, Edgar Andres Diaz Miranda, Shiying Jin, Faith Wilson, Kang An, Brooke Godbee, Xiaobin Zheng, Astrid Roshealy Brau-Rodríguez, Lei Lei

**Affiliations:** 1 Buck Institute for Research on Aging, Novato, CA 94945 USA; 2 Department of Obstetrics, Gynecology and Women’s Health, University of Missouri School of Medicine, Columbia, MO 65211 USA; 3 Division of Biological Sciences, College of Arts and Sciences, University of Missouri, Columbia, MO 65211 USA; 4 College of Health Sciences, University of Missouri, Columbia, MO 65211 USA; 5 Department of Embryology, Carnegie Institution for Science, Baltimore, MD 21218 USA; 6 Department of Medicine, Stanford University School of Medicine, Stanford, CA 94305 USA

**Keywords:** Cell biology, Physiology, Endocrinology

## Abstract

In mammalian females, quiescent primordial follicles serve as the ovarian reserve and sustain normal ovarian function and egg production via folliculogenesis. The loss of primordial follicles causes ovarian aging. Cellular senescence, characterized by cell cycle arrest and production of the senescence-associated secretory phenotype (SASP), is associated with tissue aging. In the present study, we report that some quiescent primary oocytes in primordial follicles become senescent in adult mouse ovaries. The senescent primary oocytes share senescence markers characterized in senescent somatic cells. The senescent primary oocytes were observed in young adult mouse ovaries, remained at approximately 15% of the total primary oocytes during ovarian aging from 6 to 12 months, and accumulated in aged ovaries. Administration of a senolytic drug ABT263 to 3-month-old mice reduced the percentage of senescent primary oocytes and the transcription of the SASP factors in the ovary, in addition, led to increased numbers of primordial and total follicles and a higher rate of oocyte maturation. Our study provides experimental evidence that primary oocytes, a germline cell type that is arrested in meiosis, become senescent in adult mouse ovaries and that senescent cell clearance reduced primordial follicle loss and mitigated ovarian aging phenotypes.

## Introduction

In mammalian females, the ovary produces mature eggs and steroid hormones via folliculogenesis, which are essential for supporting female fertility. In the adult ovary, ovarian folliculogenesis is sustained by a pool of primordial follicles, each containing a primary oocyte surrounded by a single layer of pre-granulosa cells^1,2^. The majority of primordial follicles remain quiescent in the adult ovary, namely the ovarian reserve, while a fraction of primordial follicles activates periodically to undergo folliculogenesis^1,3^. The number of primordial follicles decline consistently due to folliculogenesis and primordial follicle loss throughout a female’s reproductive life span. A reduced number of primordial follicles is associated with diminished ovarian function and female fertility^4–9^. Accelerated primordial follicle loss due to genetic and chromosomal abnormality (for example, Fragile X syndrome), some health conditions (for example, autoimmune diseases), and cancer treatment cause primary ovarian insufficiency/premature ovarian aging that affects at least 1% of the female population^10–14^.

Cellular senescence is linked to age-related phenotypes and diseases^15^. In tissues with active cell turnover, senescence is a cellular fate that cells undergo a stable, permanent cell-cycle arrest. Factors causing cellular senescence include telomere shortening, genomic damage, mitogens and proliferation-associated signals, epigenomic damage, and activation of tumor suppressors^16^. These factors cause and maintain senescence by two major tumor suppressor pathways—the p53/p21 and p16^INK4a^/pRB pathways^17^. Senescent cells contribute to tissue aging largely through transcriptional activation of a senescence-associated secretory phenotype (SASP)^18^. The composition of SASP varies from inflammatory cytokines, chemokines, growth factors, extracellular matrix (ECM) proteases, and bioactive lipids^19,20^. The SASP can play a beneficial and detrimental role in tissues. The SASP is involved in tissue homeostasis by facilitating recognition and clearance of senescent cells via immune responses. The SASP can also promote tissue repair and regeneration after injuries^18^. The detrimental effects of the SASP include causing inflammation, tumorigenesis, and cellular senescence in neighbor cells^21^. The most common components of the SASP include interleukin 1a, 6, and 8 (IL-1α, IL-6, and IL-8); these cytokines participate in reinforcing senescence in an autocrine and paracrine manner and are used as senescent markers^22^.

Senescence can be initiated and maintained via several pathways. Because current senescence markers do not detect the cellular processes that take place specifically in senescent cells, identification of senescent cells should be done through a careful examination of multiple senescence markers^23^. A commonly used marker is senescence-associated beta-galactosidase (SA-β-gal) detected by histochemical staining^24^. This approach measures an increased lysosomal content in senescent cells^25^. The tumor suppressor protein, p16^INK4a^ is expressed at a relatively low level in normal cells, but its expression is upregulated in senescent cells^16,26,27^. Senescent cells often display a defective nuclear envelope, which can be verified by the absence of the nuclear lamina protein lamin B1^28^. The defective nuclear lamina in senescent cells is also evident by the depletion of the high-mobility group box 1 (HMGB1) protein in the nucleus, which is one of the most abundant non-histone proteins in the mammalian cell nucleus. Extracellular HMGB1 is recognized as damage-associated molecular patterns (damps); its loss also reinforces the senescent state by chromatin regulation and RNA homeostasis of SASP-related genes^29–31^. The SASP cytokines are functional indicators of senescence caused by genomic damage or epigenomic perturbation. However, senescence growth arrest caused by the ectopic overexpression of p21 or p16^INK4a^ does not produce a SASP, despite having several other characteristics of senescent cells^15^.

In aging tissues, cells experiencing oncogenic stress may undergo either senescence or apoptosis, both cell fates lead to compromised tissue functions^17,32^. Senescent cells often resist apoptosis, thus accumulate in aging tissues and cause age-related pathologies^15,17,32–34^. Pharmacological strategies to attenuate detrimental effects of senescent cells mainly comprise two approaches—elimination of senescent cells with senolytic drugs and SASP inhibitors termed senomorphics^25^. ABT-263 “navitoclax” as a senolytic drug functions by inhibiting the activity of the antiapoptotic BCL-2 family (Bcl-2, Bcl-xL, and Bcl-w) and initiating apoptosis in senescent cells^35^. ABT-263 was shown to be beneficial to tissue functions by selectively reducing senescent cells and rejuvenating aged tissue stem cells^36,37^. Although most senescent cells are resistant to apoptosis, recent studies revealed that cellular senescence also leads to apoptosis in certain cell types. For example, senescent endothelial cells become more susceptible to apoptosis upon reduced BCL-2 or increased BAX expression, or reduced nitric oxide synthase^32,38–40^. In addition, in cultured human fibroblasts with DNA repair protein ERCC1 (excision repair cross complementing-group 1) depletion and the skin of Ercc1^−/Δ^ mice, cells become senescent initially, TNFα (tumor necrosis factor α) secreted by these senescent cells induce apoptosis in neighboring ERCC1-deficient non-senescent cells and autonomously in the senescent cells^32^.

Cellular senescence has been linked to ovarian aging and dysfunction. Ovarian stromal cells that are positive for SA-β-gal and CDKN2A (cyclin-dependent kinase inhibitor 2A) were found to accumulate in aging (8–10 months old) mouse ovaries^41^. By single-cell sequencing, a recent study found that senescent stromal cells accumulate in middle-aged human ovaries^42^. Granulosa cells can become senescent when proliferation is inhibited by Mir-200b via suppressing the expression of its target MYBL2 (MYB proto-oncogene like 2) and CDK1 (cyclin-dependent kinase 1)^43^. EIF4A3 (eukaryotic translation initiation factor 4A3) was found to induce a circRNA (circular RNA), circLRRC8A expression, and alleviate granulosa cell senescence^44^. In the premature ovarian aging mouse ovary due to the loss of primordial follicles caused by chemotherapy drugs, increased senescent granulosa cells and stromal cells that are positive for SA-β-gal were observed^45,46^.

Whether quiescent primary oocytes can become senescent and how cellular senescence in involved in primordial follicle loss and ovarian aging remain to be better characterized. In the present study, we found that a proportion of primary oocytes in primordial follicles showed features of cellular senescence in adult mouse ovaries, which we refer to as senescent primary oocytes in the present study. Senescent primary oocytes were present in young adult mouse ovaries and accumulated in aged ovaries. We also found that systemic administration of ABT263 reduced primordial follicle loss and mitigated ovarian aging phenotypes. Our study provides experimental evidence that primary oocytes, a germline cell type, can become senescent and may be involved in ovarian aging.

## Results

### Primordial follicle loss is associated with ovarian aging

**Figure 1:**
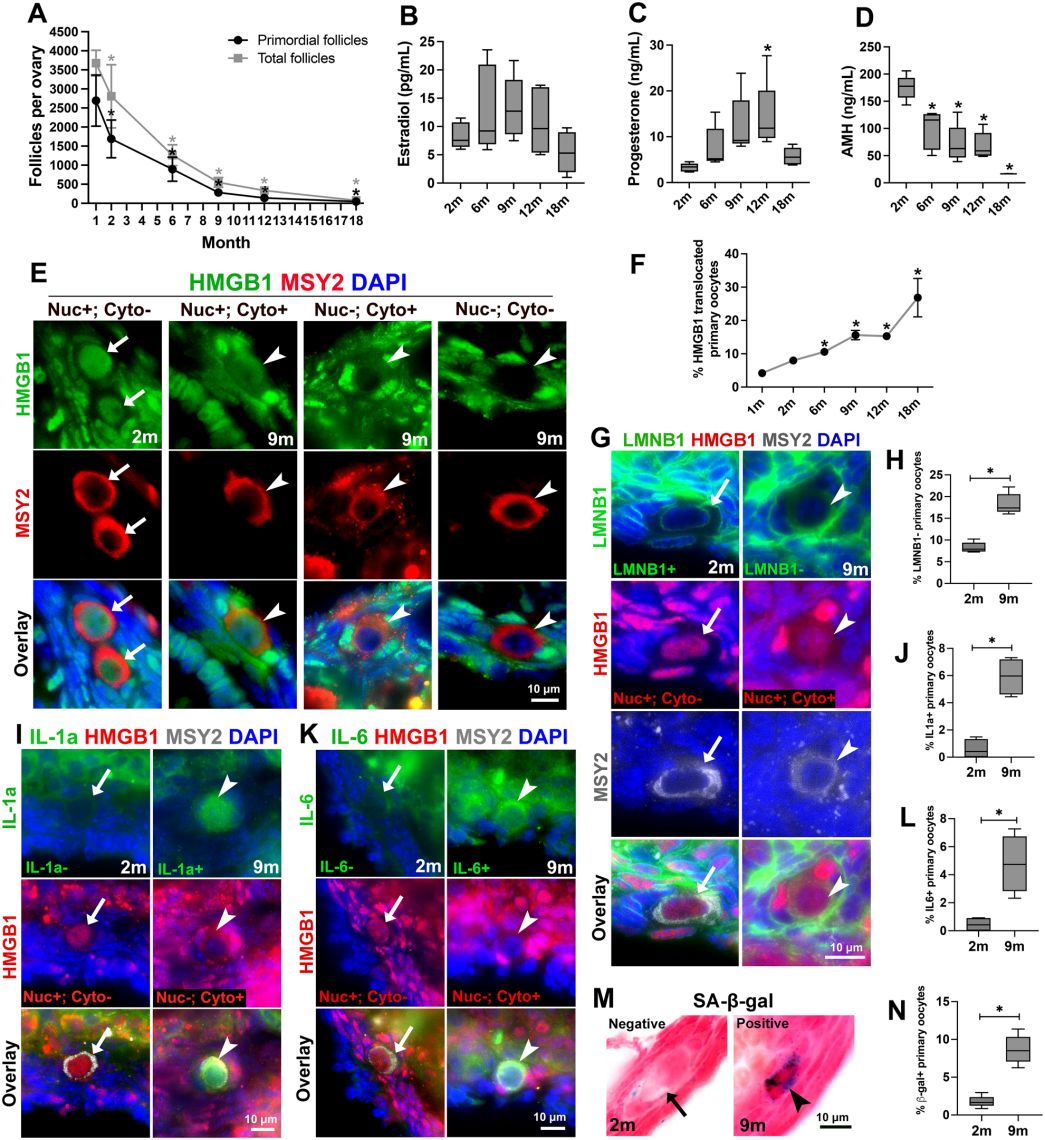
Primary oocytes with features of cellular senescence identified in adult mouse ovaries during aging. (**A**) Numbers of primordial follicles (black line) and total follicles (grey line) in the ovary declined significantly during aging. (**B**–**D**) Changes in the levels of estradiol (**B**), progesterone (**C**), and AMH (**D**) in the serum during mouse aging. (**E**) Primary oocytes stained HMGB1 positive in the nucleus and negative in the cytoplasm (arrows, nuc+; cyto−) found in 2-month (2 m) ovaries; and primary oocytes with translocated HMGB1 staining: HMGB1 positive in both the nucleus and cytoplasm (arrowheads, nuc+; cyto+), HMGB1 negative in the nucleus and positive in the cytoplasm (arrowheads, nuc−; cyto+), and HMGB1 negative in both the nucleus and cytoplasm (arrowheads, nuc−; cyto−). (**F**) The percentage of primary oocytes with translocated HMGB1 increased during mouse aging. (**G**) The primary oocyte that was nucleus-positive (Nuc+; Cyto) for HMGB1 stained positive for nuclear envelope protein lamin b1 (arrows, LMNB1+); the primary oocyte had translocated HMGB1 staining (Nuc+; Cyto+) stained negative for lamin b1 (arrowheads, LMNB1−). (**H**) The percentage of primary oocytes had LMNB1 negative staining in 2-month and 9-month ovaries. (**I**) The primary oocyte that was only nucleus-positive for HMGB1 stained negative for IL-1α (IL-1α−; arrows); the primary oocyte had translocated HMGB1 staining (Nuc−; Cyto+) stained positive for IL-1α (IL-1α+; arrowheads). (**J**) The percentage of primary oocytes had IL-1α positive staining in 2-month and 9-month ovaries. (**K**) The primary oocyte that was nucleus-positive for HMGB1 stained negative for IL-6 (IL-6−; arrows); the primary oocyte with translocated HMGB1 staining (Nuc−; Cyto+) stained positive for IL6 (IL-6+; arrowheads). (**L**) The percentage of primary oocytes had IL-6-positive staining in 2-month and 9-month ovaries. (**M**) Primary oocyte with SA-β-gal negative staining (arrow) and positive staining (arrowhead). (**N**) The percentage of primary oocytes had SA-β-gal positive staining in 2-month and 9-month ovaries. Data in the graph are presented as mean ± SD. *Represents significant difference. n = 5 mice at each time point were analyzed in all the graphs.

To reveal the timing of primordial follicle loss and how it is associated with diminished ovarian function, we quantified the number of follicles at different stages of folliculogenesis in the ovaries of mice at ages of 1 month, 2 months, 6 months, 9 months, 12 months, and 18 months. Follicles were staged based on established morphological standards and quantified based on previously published protocols using consecutive sections of an entire ovary^47–49^. Numbers of primordial, transitional (the intermediate stage between primordial and primary follicles), primary, secondary, preantral, and antral follicles in each ovary were quantified. We found that from 1 to 18 months, the numbers of primordial and total follicles declined in a similar trend. On average, an ovary lost 66.8% of the primordial follicles and 65.7% of the total follicles from 1 to 6 months. By 12 months, when the majority of the female mice lose their fertility, only 5.2% of the primordial follicles and 9.1% of the total follicles of a 1-month-old ovary remained. This result suggests that primordial follicle depletion is the primary cause of follicle loss and declined ovarian function during ovarian aging (Fig. 1A).

To further make a connection between follicle number and ovarian hormone production, we measured the levels of estradiol, progesterone, and AMH (anti-Mullerian hormone) in the serum of mice at the ages of 2 months, 6 months, 9 months, 12 months, and 18 months. On average, estradiol level peaked at 9 months and declined afterward, but no significant difference was observed between the mice at different ages (Fig. 1B). Progesterone levels increased as mice aged and were the highest at 12 months, which was significantly higher than that in 2-month-old mice (Fig. 1C). We observed a wide range of variation in hormone levels, which may be due to blood sample collection without examining estrous cycles of the mice. This is a significant limitation of this study. Consistent with a previously identified connection between AMH level and total follicle numbers^50^, the level of AMH was the highest at 2 months and declined significantly as mice aged (Fig. 1D).

### Primary oocytes with features of cellular senescence identified in adult ovaries

To investigate whether or not ovarian cells become senescent during aging, we first carefully examined the expression and location of HMGB1 in the adult mouse ovary. In 2-month-old mouse ovaries, HMGB1 was located in the nucleus of granulosa cells, thecal cells, and interstitial stromal cells (Supplementary Fig S1). The majority of the primary oocytes in primordial follicles had HMGB1 protein exclusively in the nucleus (Fig. 1E, arrows, Nuc+; Cyto−). However, we also observed a fraction of primary oocytes that showed translocated HMGB1 (Fig. 1E, arrowheads), including: (1) HMGB1 positive staining in both the nucleus and cytoplasm (Nuc+; Cyto+; (2) HMGB1 negative staining in the nucleus but positive in the cytoplasm (Nuc−; Cyto+); and (3) HMGB1 negative staining in both the nucleus and cytoplasm (Nuc−; Cyto−). Because primary oocytes that stained HMGB1 negative in both the nucleus and cytoplasm showed normal histological structure, stained positive for oocyte marker MSY2 as the rest of the primary oocytes, and were negative for apoptotic markers (cleaved caspase-3 and TUNEL, Supplementary Table S1), we considered these primary oocytes as a subtype of translated HMGB1, instead of dying oocytes. Primary oocytes with translocated HMGB1 were observed starting in young (1 month) ovaries at the rate of 4.17%, remained approximately 15% during ovarian aging (from 6 to 12 months), and increased to 26.8% in aged ovaries by 18 months, in which there were 47 ± 14 primordial follicles per ovary on average (Fig. 1F). Since most female mice have lost their fertility around 18 months, this observation indicates that primordial follicles with senescent primary oocytes may have lost their potential for folliculogenesis, although morphologically indistinguishable from dormant primordial follicles.

To further characterize whether the primary oocytes with translocated HMGB1 also have other features of cellular senescence, we examined the expression of nuclear envelope protein lamin B1 and SASP cytokines IL-1α and IL6 in the primary oocytes. We found that primary oocytes with positive lamin B1 staining had HMGB1 staining just in the nuclei (Nuc+; Cyto−, Fig. 1G, arrows), and primary oocytes that have translocated HMGB1 staining (Nuc+; Cyto+ in Fig. 1G, and Nuc−; Cyto− in Supplementary Fig. S2A, arrowheads) stained negative for lamin B1 (Fig. 1G, arrowheads). From 2 to 9 months, on average the percentage of lamin B1-negative primary oocytes increased from 8.2 to 18.3% (Fig. 1H). We found that primary oocytes with translocated HMGB1 (Nuc−; Cyto + in Fig. 1I and Nuc−; Cyto− in Supplementary Fig S2B, arrowheads) were associated with positive staining of IL-1α. And similarly, primary oocytes with translocated HMGB1 (Nuc−; Cyto+ in Fig. 1K and Nuc−; Cyto− in Supplementary Fig S2C, arrowheads) were associated with positive staining of IL-6. In 2-month-old ovaries, on average 0.58% of the primary oocytes are IL-1α positive, and 0.44% are IL6 positive (Fig. 1J). In 9-month-old ovaries, 5.9% of the primary oocytes are IL-1α positive, and 4.8% are IL-6 positive (Fig. 1J and L). All primary oocytes that were lamin B1 negative, IL-1α or IL-6 positive had translocated HMGB1. In addition, we also found that some primary oocytes stained positive for SA-β-gal (Fig. 1M, arrowhead). The average percentage of SA-β-gal-positive primary oocytes increased from 1.8% at 2 months to 8.7% by 9 months (Fig. 1N). Taken together, through examining several well-established markers of cellular senescence, we found that primary oocytes with translocated HMGB1 were also positive for at least one other senescence marker, indicating that these primary oocytes had cellular features of senescence. Based on these results, we refer primary oocytes with translocated HMGB1 as senescent primary oocytes in the present study.

To elucidate the expression of p21 and p16^INK4a^ in adult mouse ovaries, we conducted antibody staining of p21 in 6-month-old C57BL/6J ovaries, and of luciferase in 6-month-old p16-3MR mouse ovaries, in which p16^INK4a^ promoter drives the expression of 3MR (trimodality reporter) fusion protein, including luciferase^51^. We found that p21 is expressed in all cell types in the ovary. All primary oocytes in the p16-3MR mouse ovary, regardless of their HMGB1 location, were positive for luciferase (Supplementary Fig S3). These results suggest that p21 and p16^INK4a^ may not be used to identify senescent primary oocytes in adult mouse ovaries.

### ABT263 administration reduced senescent primary oocytes

**Figure 2:**
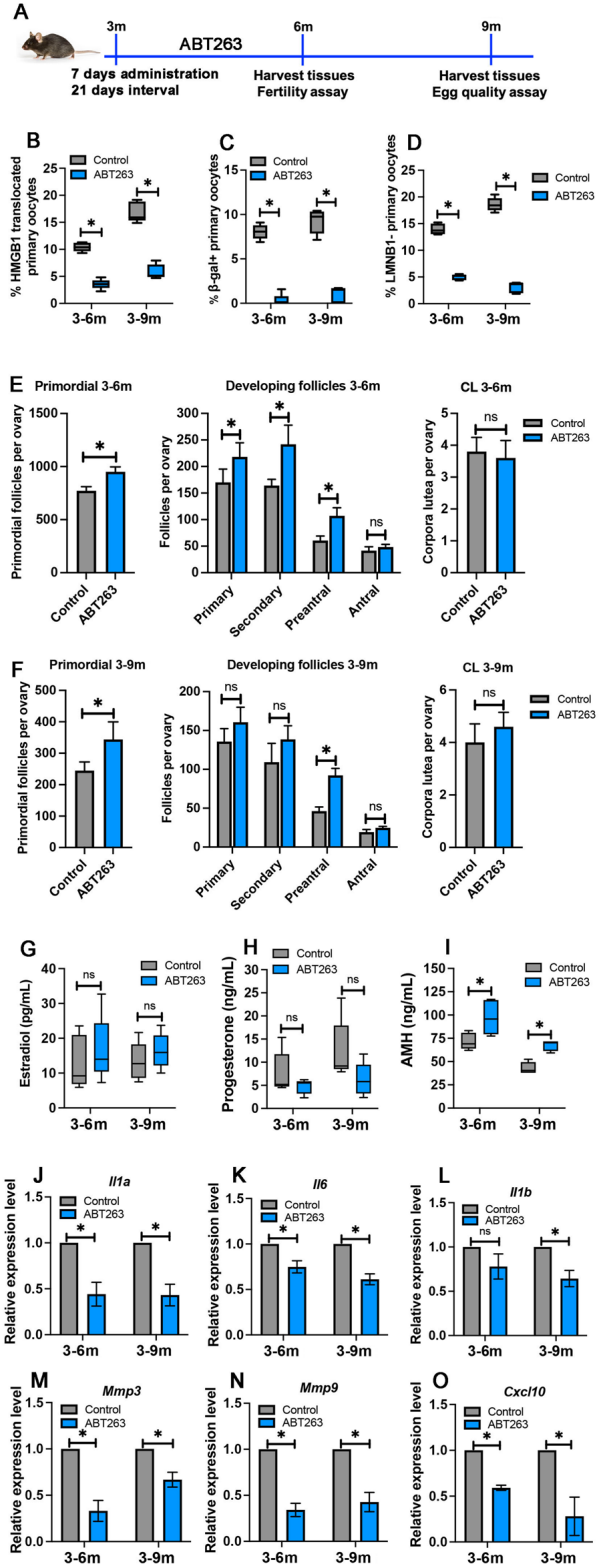
Senolytic drug ABT263 treatment mitigated ovarian aging phenotypes. (**A**) Schematic timeline of ABT263 treatment. (**B**–**D**) Percentages of HMGB1 translocated primary oocytes (**B**), β-gal-positive primary oocytes (**C**), and LMNB1-negative primary oocytes (D) in control and ABT263-treated ovaries at 6 months and 9 months. (**E**, **F**) Number of primordial follicles, developing (primary, secondary, preantral, and antral) follicles, and corpora lutea in control and ABT263-treated ovaries at 6 months (**E**) and 9 months (**F**). (**G**–**I**) Levels of estradiol (**G**), progesterone (**H**), and AMH (**I**) in the serum of control mice and ABT263-treated mice at 6 months and 9 months. (**J**–**O**) Relative expression of mRNAs of *Il1a* (**J**), *Il1b* (**K**), *Il6* (**L**), *Mmp3* (**M**), *Mmp9* (**N**), and *Cxcl10* (**O**) in control and ABT263-treated ovaries at 6 months and 9 months. Data in the graph are presented as mean ± SD. *Represents significant difference; ns represents no significant difference. n = 5 mice at each time point were analyzed in graphs B-I. Three independent biological replicates were conducted in graphs (**J**–**O**).

To elucidate potential roles of senescent cells in ovarian aging, ABT263, a senolytic drug that selectively causes cell death in senescent cells was administrated to adult (3-month-old) C57BL/6J mice via oral gavage for 3 months^36^. At the end of the 3-month treatment, the ovaries of control mice and ABT-treated mice (now at 6 months of age) were collected for analyses. Control and ABT-treated mice were also housed with male mice for fertility assay for 3 months, and mouse ovaries (now at 9 months of age) were collected for analyses at the of the fertility assay (Fig. 2A).

After a 3-month ABT263 administration, the percentage of HMGB1 translocated primary oocytes decreased significantly in both 6 months (10.4% in control ovaries vs. 3.6% in ABT263-treated ovaries) and 9 months ovaries (16.9% in control ovaries vs. 5.9% in ABT263-treated ovaries) (Fig. 2B). Similarly, ABT263 treatment significantly reduced the percentage of SA-β-gal-positive primary oocytes in the ovary of 6 months (8.0% in control ovaries vs. 0.3% in ABT263-treated ovaries) and 9 months (9.2% in control ovaries vs. 1.0% in ABT263-treated ovaries) (Fig. 2C); and lamin B1-negative primary oocytes in the ovary of 6 months (14.0% in control ovaries vs. 5.0% in ABT263-treated ovaries) and 9 months (18.6% in control ovaries vs. 3.1% in ABT263-treated ovaries) (Fig. 2D). These results demonstrated that ABT263 treatment reduced the number of senescent primary oocytes.

### ABT263 administration mitigated ovarian aging phenotypes

Ovarian aging refers to declined ovarian functions as animals age^5^. In the present study, we investigated the following phenotypes that are previously characterized in aging ovaries: reduced primordial follicle number, decreased hormone production, increased inflammation, and declined rate in oocyte maturation and oocyte quality^5,52–55^.

To assess the effect of ABT263 treatment on primordial follicle loss, we first compared the numbers of primordial and total follicles between the control and ABT-treated ovaries. We found that at both 6 months (right after 3 months of ABT263 treatment) and 9 months (6 months after the ABT263 treatment), a significantly increased number of primordial follicles were found in ABT263-treated ovaries (6-months: 951 ± 46 primordial follicles per ovary and 9 months: 343 ± 56 primordial follicles per ovary) compared to those in control mice (6-month: 772 ± 38 primordial follicles per ovary and 9 months: 244 ± 27 primordial follicles per ovary) (Fig. 2E and F). There were more preantral follicles observed in ABT263-treated ovaries, but comparable numbers of antral follicles and corpora lutea in ABT263-treated and control ovaries (Fig. 2E and F). This result indicates that ABT263 treatment may contribute to a slightly increased primordial follicle recruitment without causing a reduced number of primordial follicles, suggesting that increased primordial follicle numbers in ABT263-treated ovaries may primarily result from a reduced primordial follicle loss.

Compared to control mice, ABT263 treatment led to increased levels of estradiol and decreased levels of progesterone without statistical significance at both 6 months and 9 months of age (Fig. 2G and H). Consistent with increased numbers of primordial and developing follicles, ABT263 treatment caused a significantly increased level of AMH in both 6-month and 9-month-old mice (Fig. 2I).

To evaluate the effect of ABT263 treatment on the production of SASP cytokines in the ovary, we examined mRNA expression of *Il1a*, *Il1b*, *Il6*, *Mmp3* (matrix metallopeptidase 3), *Mmp9*, and *Cxcl10* (C-X-C motif chemokine ligand 10) by real-time PCR. Consistent with a decreased number of senescent primary oocytes, ATB263 treatment reduced the expression of these cytokines in both 6-month and 9-month-old mouse ovaries (Fig. 2J–O). Taken together, these results suggest that ABT263 treatment mitigated ovarian aging by reducing the rate of primordial and total follicle loss and SASP production in the ovary.

### ABT263 administration improved egg quality 

**Figure 3:**
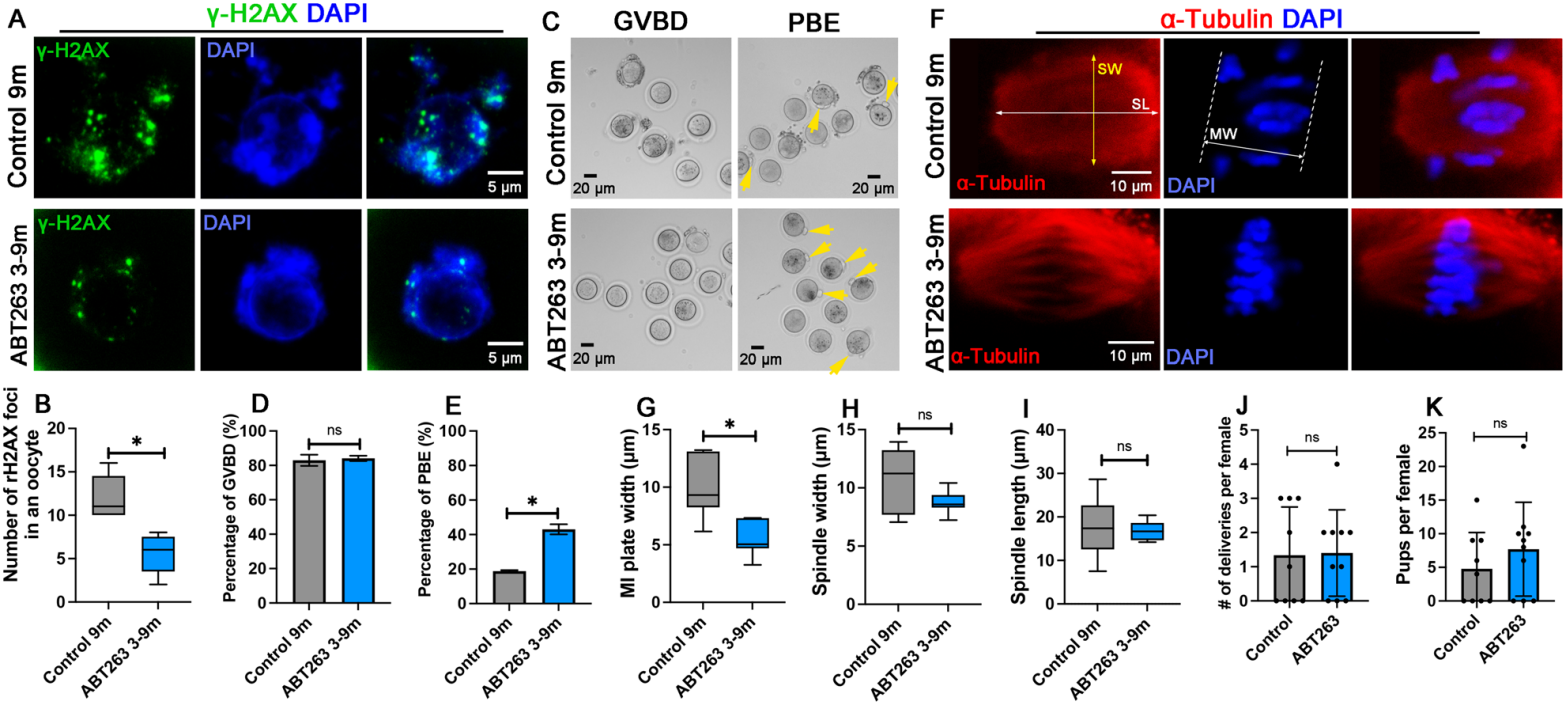
Effect of ABT263 treatment on oocyte maturation and fertility. (**A**) Oocytes from 9-month-old control mice (control 9 m) and ABT263-treated mice (ABT263 3-9 m) were stained with an antibody to γ-H2AX to detect DNA damage. DNA was revealed by DAPI staining. (**B**) Numbers of γ-H2AX-positive foci in the oocytes isolated from control ovaries and ABT263-treated ovaries at 9 months. (**C**) Brightfield microscopic images showing that oocytes isolated from control ovaries and ABT263-treated ovaries at 9 months underwent germinal vesicle breakdown (GVBD), and more oocytes isolated from ABT263-treated ovaries completed first polar body extrusion (PBE, arrows). (**D**, **E**) Percentages of oocytes underwent GVBD (**D**) and PBE (**E**). (**F**) Examples of meiotic spindles observed in oocytes isolated from control ovaries and ABT263-treated ovaries at 9 months. (**G**–**I**) The width of the metaphase I plate (MW) (**G**), the width of the meiotic spindle (SW) (**H**), and the length of the meiotic spindle (SL) (**I**) in the oocytes isolated from control ovaries and ABT263-treated ovaries at 9 months. (**J**–**K**) Number of deliveries (J) and number of pups (**K**) of each control mouse and ABT263-treated mouse during fertility assay. Data in the graph are presented as mean ± SD. *Represents significant difference; ns represents no significant difference. Three experimental replicates (n = 5 mice in each replicate) were conducted for analyses in graphs (**B**–**I**). n = 10 mice from the control and ABT-treated groups were used for fertility assay in graphs (**J**) and (**K**).

To evaluate the effect of ABT263 treatment on egg quality in aging mice, oocytes were collected from 9-month-old ATB263-treated and control mice for antibody staining and oocyte maturation experiments. Oocytes with a germinal vesicle (GV) representing an intact nucleus were fixed and stained with an antibody to γ-H2AX that labels DNA double-strand breaks. We found a significantly decreased number of DNA double-strand breaks shown by γ-H2AX-positive foci in the nucleus of the oocytes from ATB263-treated mouse ovaries (Fig. 3A, B). To assess oocyte maturation, oocytes at the GV stage were cultured for 2 h to observe their ability to resume meiosis represented by germinal vesicle breakdown (GVBD) and for 14 h to observe their ability to complete meiosis represented by first polar body extrusion (PBE). We found that a similar percentage of oocytes from control mice (82.9%) and ABT263-treated mice (84.1%) were able to undergo GVBD (Fig. 3C and D). However, a significantly higher rate (43.1%) of oocytes from ABT263-treated mice were able to complete meiosis I during oocyte maturation compared with that (18.8%) of the oocytes from control mice (Fig. 3C and E). We further analyzed spindle morphology and found that oocytes from control mice had a wider metaphase plate compared to ABT263-treated mice, suggesting a better chromosomal alignment in oocytes from ABT263-treated mice (Fig. 3F and G). Although greater variations in the width and length of the spindles were observed in oocytes from control mice, there were no significant differences between the oocytes from control and ABT263-treated mice (Fig. 3F, H, I). Consistent with the results of oocyte meiotic maturation, the fertility assay from 6 to 9 months of age revealed that ABT263-treated mice had a similar number of deliveries, but reproduced more pups than that of control mice (ABT263-treated: 5 ± 5 pups per litter; control: 8 ± 7 pups per litter) (Fig. 3J and K). These results suggest that ABT263 administration improved oocyte quality, and, to a limited extent, female fertility.

### Changes in transcriptomes in aging and ABT263-treated ovaries

**Figure 4:**
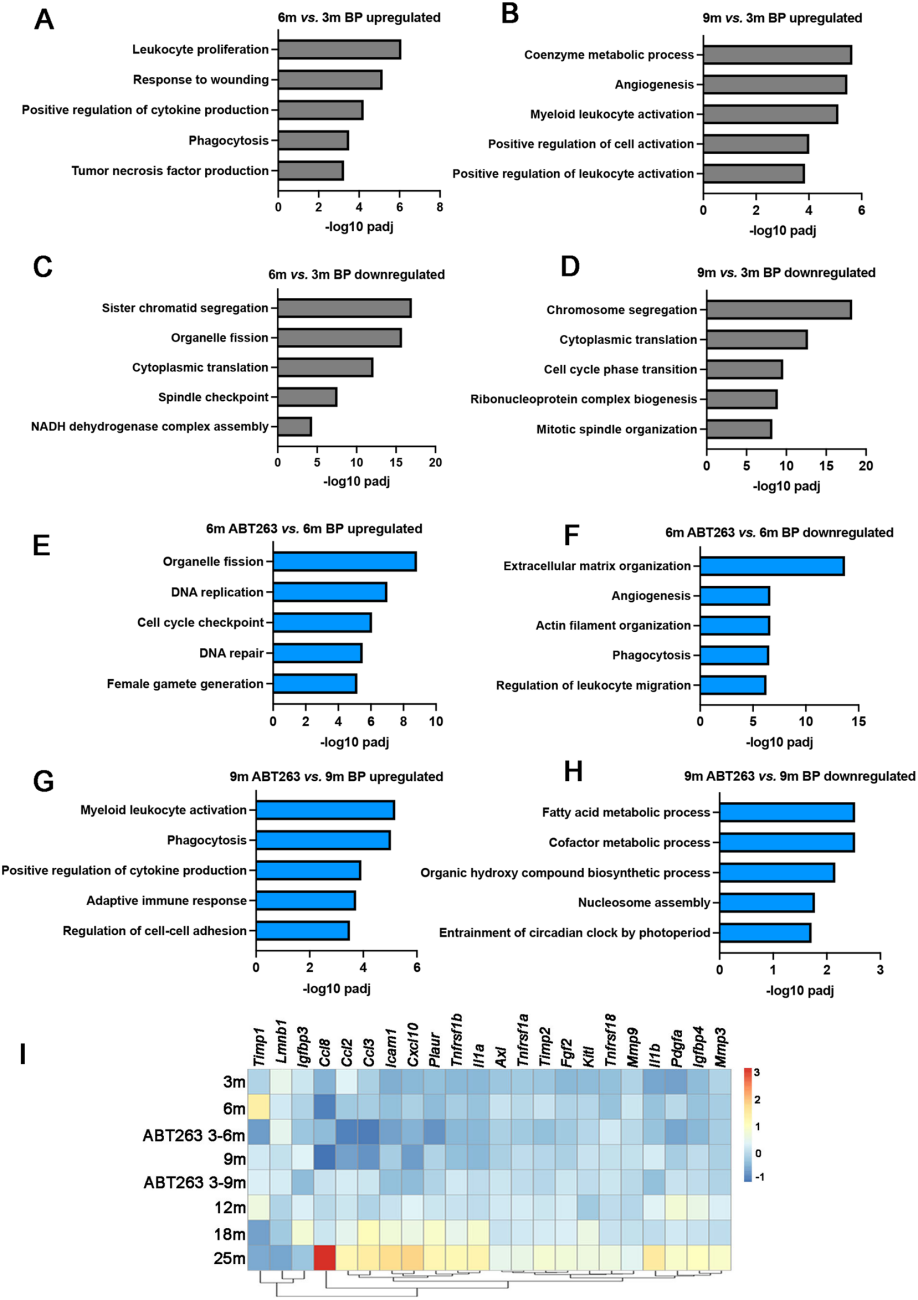
Comparative transcriptome profiling of mouse ovaries during aging and between the ovaries of ABT263-treated mice and control mice. (**A**–**D**) Gene ontology analysis of biological pathways (BP) that were significantly upregulated in the ovaries of 6-month-old mice (**A**) and 9-month-old mice (**B**) compared with 3-month-old mice; and the BPs that were significantly downregulated in the ovaries of 6-month-old mice (**C**) and 9-month-old mice (**D**) compared with 3-month-old mice. (**E**, **F**) BPs that were significantly upregulated (**E**) or downregulated (**F**) in 6-month-old ovaries of ABT263-treated mice compared with that of control mice. (**G**, **H**) BPs that were significantly upregulated (**G**) or downregulated (**H**) in 9-month-old ovaries of ABT263-treated mice compared with that of control mice. (**I**) Heatmap showing fold change in mRNA expression of SASP factors in mouse ovaries during aging and between the ovaries of ABT263-treated mice and control mice. n = 3 mice at each time point were used for RNA extraction and sequencing.

To reveal potential mechanisms of how cellular senescence may contribute to ovarian aging, we conducted mRNA sequencing of mouse ovaries at different ages (3 months, 6 months, 9 months, 12 months, 18 months, and 25 months), and the ovaries of the mice treated with ABT263, or control vehicle as shown in Fig. 2A (Supplementary Tables S2–5). Firstly, compared with 3-month-old ovaries, both 6-month and 9-month ovaries showed increased expressions in inflammation-related biological pathways, including leukocyte proliferation/activation, cytokine production, and TNF production (Fig. 4A and B). Both 6-month and 9-month ovaries had decreased expressions in biological pathways involved in organelle fission, meiosis (sister chromatid/chromosome segregation, spindle checkpoint/organization, cell cycle phase transition), translation regulation (cytoplasmic translation, ribonucleoprotein complex biogenesis) and mitochondrial function (NADH dehydrogenase complex assembly). These results indicate potential causes of declined oocyte quality and female fertility (Fig. 4C and D).

When comparing the 6-month-old ovaries of control mice with the ovaries of ABT263-treated mice, ABT263-treated ovaries had increased expressions in biological pathways of organelle fission, meiosis (DNA replication, cell cycle checkpoint, and DNA repair) (Fig. 4E); and decreased expressions in extracellular matrix organization, angiogenesis, actin filament organization, phagocytosis, and regulation of leukocyte migration (Fig. 4F). At 9 months, compared with control ovaries, ABT263-treated ovaries had an increased expression in immune response-related biological pathways, including myeloid leukocyte activation, phagocytosis, positive regulation of cytokine production, and adaptive immune response (Fig. 4G); and a decreased expression in pathways involved in metabolism (fatty acid metabolic process, cofactor metabolic process, and organic hydroxy compound biosynthetic process), nucleosome assembly, and entrainment of circadian block by photoperiod (Fig. 4H). These results suggest that ABT263 treatment mitigated ovarian aging at the transcription level (Fig. 4).

We further conducted comparative profiling on the mRNA expression of the SASP factors between ABT263-treated mice and control mice, as well as during ovarian aging (Fig. 4I). We found that compared with young ovaries (3 months), the expression of SASP factors increased during ovarian aging from 6 to 12 months and to a greater extent in aged (18 months and 25 months) ovaries; and ABT263 treatment selectively reduced the expression of some SASP factors. From 3 to 6 months, mRNA of SASP factors *Timp1* (TIMP metallopeptidase inhibitor 1), *Icam1* (intercellular adhesion molecule 1)*, cxcl10 (C-X-C motif chemokine ligand 10), Tnfrsf1b (*TNF receptor superfamily member 1b*)*, *Axl (AXL receptor tyrosine kinase), Timp2 (TIMP metallopeptidase inhibitor 2), Fgf2 (fibroblast growth factor 2), Kitl (KIT ligand), Mmp9, Il1b, Pdgfa* (platelet-derived growth factor a) showed an increased expression. In the 6-month-old ABT263 treated ovaries, expressions of *Timp1*, *Ccl2 (*C-C motif chemokine ligand 2*)*, *Ccl3, Icam1, cxcl10, Plaur* (plasminogen activator, urokinase receptor), *Tnfrsf1b, Axl, Timp2, Fgf2, Mmp9, Pdgfa* were downregulated due to ABT263 administration. Compared with 3 months ovaries, 9 months ovaries had upregulated expressions in *Timp1, Igfbp3* (insulin-like growth factor binding protein 3), *Icam1, Plaur, Tnfrsf1a, Kitl, Tnfrsf18 (TNF receptor superfamily member 18), Il1b, Pdgfa, Igfbp4.* And the ABT263 treatment reduced the expression of *Igfbp3, Kitl,* and *Il1b* (Fig. 4I)*.* The expression of the SASP factors increased significantly in the ovaries of 18 and 25 months when most follicles have been depleted, indicating that in the aged ovaries, non-follicular somatic cells may become the major cell types that produce the SASP, and senescent cell clearance might be severely compromised. Although the changes in whole ovary transcriptome indicate upregulated inflammatory pathways, how each cell type changes in SASP factor expression and the associated functions during ovarian aging should be carefully examined in future studies.

## Discussion

Quiescent primordial follicles are recognized by their morphological structure of a primary oocyte at ~ 20 μm in diameter surrounded by a single layer of squamous pregranulosa cells. In the present study, we demonstrated that although morphologically undistinguishable, some primary oocytes in the primordial follicle had features of cellular senescence, including positive for SA-β-gal staining, translocated HMGB1 due to defective nuclear envelopes and most importantly produced the SASP cytokines (IL-1α and IL-6) that could cause local inflammation and tissue destruction. It is worth noting that the percentages of IL-1α -positive, IL-6-positive, and SA-β-gal-positive primary oocytes were lower than the percentage of primary oocytes with translocated HMGB1 (Fig. 1F, H, J, L and N). This result indicates that primary oocytes with defective nuclear envelopes (as indicated by the lack of laminb1) and translocated HMGB1 may present primary oocytes at the stage of becoming senescent. The primary oocytes with the SASP production (IL-1α or IL-6) are SA-β-gal positive due to an increased lysosomal content may represent primary oocytes that have become senescent completely. These results demonstrated that senescent primary oocytes, as germline cells that are arrested at meiotic prophase I, share a common cellular feature of cellular senescence observed in somatic cells. Our observation complements previous findings that terminally differentiated cells, including neurons, cardiomyocytes, and adipocytes, can become senescent^25^. Our study suggests that similar to apoptosis and quiescence, cellular senescence is a general cellular state that can take place not only in mitotic and non-mitotic cells but also in germ cells in meiosis.

In somatic cells, senescence can be triggered through two pathways. Firstly, persistent DNA damage activates the p53 tumor suppressor, which induces the expression of cell cycle inhibitor p21 and causes cell cycle arrest. When experiencing stress that does not involve direct DNA damage, increased p16^INK4a^ expression activates pRB tumor suppressor, which silences certain proliferative genes by heterochromatinization^15^. In quiescent primary oocytes, TAp63α, a p53 ortholog, instead of p53 is highly expressed and is in a closed, inactive dimeric conformation^56,57^. Upon DNA damage caused by radiation or chemotherapy drugs, ATM (ataxia telangiectasia mutated kinase) or ATR (ATM and rad3-related) activates checkpoint kinase 2 (CHK2) or checkpoint kinase 1 (CHK1). CHK1/2 further phosphorylates TAp63, which activates its downstream targets to initiate apoptosis in primary oocytes^57–63^. In the ovary under physiological aging, DNA damage takes place less extensively compared with that induced by radiation or chemotherapy. Whether primary oocytes undergo senescence first before undergoing apoptosis will be addressed in our future study. In addition to DNA damage, whether increased oxidative stress due to dysfunctional mitochondria or telomere shortening in primary oocytes causes primary oocyte senescence is an intriguing open question that will be further investigated in our future study^25,64,65^.

Because most senescent cells are resistant to apoptosis, the accumulation of senescent cells in the tissue during aging could cause detrimental effects. In the present study, we found that although the number of primordial follicles declined significantly from 2 months (1687 ± 498 per ovary) to 9 months (284 ± 72 per ovary), the percentage of senescent primary oocytes increased from 7.97 ± 0.18% to 15.6 ± 1.41%, this indicates that senescent primary oocytes may not be resistant to cell death, instead, senescent primary oocytes may be constantly lost in the adult ovary^17^. This observation is consistent with the new role of cellular senescence in causing apoptosis in senescent cells autonomously and neighboring cells through the SASP^32^. Previous studies found that several cytokines, including interleukin 16 (IL-16), IL-6, IL-18, and TNFα cause primordial follicle loss or activation^54,66,67^. We found that senescent primary oocytes produced IL-1α and IL-6; whether these two SASP cytokines cause primary oocyte loss will be investigated in our future study. The possible effect of senescent primary oocytes on primordial follicle loss was further supported by ABT263 administration, which caused a decreased percentage of senescent primary oocytes. We found increased numbers of primordial and total follicles in ABT263-treated mouse ovaries compared with control ovaries at 6 months and 9 months (Fig. 2), suggesting that a decreased senescent primary oocyte accumulation is associated with reduced primordial follicle loss in adult mouse ovaries.

ABT-263 initiates apoptosis in senescent cells through inhibiting the activity of the antiapoptotic BCL-2 family, and caused senescent primary oocyte clearance and a decreased primordial follicle loss in the present study^35^. Although we did not find apoptotic primary oocytes (cleaved caspase-3 or TUNEL positive) during natural aging in 2-month and 9-month-old mouse ovaries (Supplementary Table S1), whether or not senescent primary oocyte clearance in ABT263-treated ovaries took place via apoptosis should be carefully examined in future study. However, our results are consistent with previous findings that BCL2 is involved in primordial follicle loss. In the mouse models with Bcl-2 over expression in mouse germ cells or oocytes, an increased number of primordial follicles were observed in newborn ovaries and primordial follicles were resistant to cell death compared to that in wildtype mice^68,69^. Our results suggest that compared with quiescent primary oocytes, senescent primary oocytes might be more prone to apoptosis upon inhibited BCL-2 activity.

Because ABT263 was administrated systemically in the present study, senescent cell clearance also took place in other tissues, which may have caused reduced inflammatory cytokine production systemically. Thus, reduced primordial follicle loss observed in ABT263-treated mouse ovaries may be attributed by other unidentified factors, in addition to an increased senescent primary oocyte clearance identified in the present study. We speculate that transient accumulated senescent primary oocytes at ~ 15% in ovaries from 3 to 6 months during normal aging may lead to senescence in neighboring primary oocytes and trigger a continued senescence-associated primary oocyte loss that causes the onset of ovarian aging.

Although a relatively low percentage of senescent primary oocytes remained in mouse ovaries during ovarian aging up to 12 months, the transcriptome profile of adult ovaries in our study revealed that SASP-related inflammatory biological processes (BP) were upregulated from 3 to 6 months and remained upregulated in 9 months ovaries, including TNF and cytokine production, and leukocyte proliferation and activation. This result indicates that immune-response in the adult ovary is active before and during ovarian aging, which may play a role in senescent cell clearance (Fig. 4A). The BPs that were downregulated during aging are mostly involved in cell cycle, DNA repair, and spindle checkpoint and organization, consistent with increased DNA damage and decreased oocyte meiotic maturation and female fertility observed in present and many previous studies. It is worth noting that some pro-inflammatory cytokines, for example, IL-17 and TNFα directly or indirectly affect cytoskeleton organization^70,71^, which plays an essential role in oocyte meiotic maturation. Our results indicate that an increased inflammatory response in the adult ovary before and during ovarian aging may contribute to decreased oocyte meiotic maturation and increased aneuploidy in mature oocytes. This hypothesis is supported by our results from the ABT263 treatment experiment—a reduced immune-response/senescence-associated inflammation and improved oocyte maturation and fertility were observed in ATB263-treated ovaries. Thus, reducing senescence-associated inflammatory response may present a potential clinical approach to improve female fertility. What are the initial cells that trigger inflammation and cause the onset of ovarian aging? Our study argues that SASP-producing senescent primary oocytes observed starting in young mouse ovaries (1–2 months), maintained during ovarian aging (6–12 months), and accumulated in aged mouse ovaries (18 months), may be the initial cellular source triggering senescent-associated inflammation in ovarian somatic cells, which often observed in aged mouse ovaries^41^.

In summary, we reported in the present study that some quiescent primary oocytes become senescent starting in young mouse ovaries, maintained during ovarian aging, and accumulate in aged ovaries. Senescent cell clearance reduced primordial follicle loss and senescence-associated inflammation in the adult ovary, and improved oocyte maturation and female fertility. Our study suggests that cellular senescence is involved in ovarian aging and speculates that SASP-producing senescent primary oocytes may be the initial cellular source triggering senescent-associated inflammation and the onset of ovarian aging.

## Materials and methods

### Animals

All animal experiments were approved by the Institutional Animal Care & Use Committee (IACUC) at the University of Missouri (protocol number: 36647) and the Buck Institute for Research on Aging (protocol number: A10207), and conducted in accordance with the Guide for the Care and Use of Laboratory Animals of the University of Missouri, the Standard Operating Procedure for Animal Research of the Buck Institute, and the ARRIVE guidelines. C57BL/6J (000664) mice and p16-3MR mice (037045) were acquired from the Jackson Laboratory. The carbon dioxide (CO_2_) inhalation system in the vivarium facility was used for euthanasia. During euthanasia, the mice were placed into a chamber, in which CO2 was added slowly to increase its concentration. The personnel performing euthanasia were required to monitor the process and wait for at least one minute after no movement, visible inhaling, and heartbeat was detected. An approved secondary method was used to ensure the death of the mice.

### Antibody staining and follicle quantification

Adult ovaries were fixed in 4% paraformaldehyde (PFA) overnight at 4 °C. After fixation, ovaries were washed in phosphate-buffered saline (PBS) and incubated in 30% sucrose overnight before embedding in optimal cutting temperature (OCT) compound. For quantifications of total follicles and senescent primary oocytes, serial frozen sections were cut at 10 µm of the entire ovary and number of the total sections of each ovary was recorded. Frozen sections at every fifth interval were collected and stained with antibodies to HMGB1 and MSY2 for follicle and senescent primary oocyte quantifications. Follicles at different stages of folliculogenesis were staged based on established morphological characteristics used in previously published studies^47–49^. Follicles were counted in all stained sections. The numbers of follicles at the following stages of folliculogenesis (primordial, transition, primary, secondary, preantral, and antral) per ovary were calculated as the number of follicles in each ovary = follicles/section × total sections of each ovary. Primary oocytes of primordial follicles were recognized by MSY2-positive staining. HMGB1 translocated primary oocytes were defined as MSY2-positive primary oocytes enclosed in primordial follicles that are: HMGB1 nucleus-positive and cytoplasmic-positive; nucleus-negative and cytoplasmic-positive; and nucleus-negative and cytoplasmic-negative. The number of senescent primary oocytes in each ovary = senescent primary oocytes/section x total sections of each ovary. For quantifications of primary oocytes that were IL-1α-positive, IL-6-positive, LMNB1-negative or β-gal-positive, frozen sections at every fifth interval of the ovary were collected, and 12 sections from the middle of the ovary were stained with antibodies to HMGB1, MSY2, and LMNB1 (or IL-1α, or IL-6), or stained with the β-galactosidase staining kit. All primary oocytes on these sections were examined and quantified. For the quantification of apoptotic primary oocytes, frozen sections at every fifth interval of each ovary were collected, 12 sections from the middle of the ovary were stained with the TUNEL apoptosis detection kit, and 20 sections from the middle of the ovary were stained with antibodies to cleaved caspase-3. All primary oocytes on these sections were examined and quantified. Please see Supplementary Table S6 for detailed information of antibodies and staining kits.

### Serum collection and hormone measurement

Blood was collected from each mouse via cardiac puncture using a 1 ml syringe with a 21G needle immediately after the mouse was euthanized. The blood was placed in a 1.5 ml microcentrifuge tube and sat on the tube rack at room temperature for 30 min followed by 4 degrees overnight to allow the blood to clot. The serum was isolated by centrifuging the blood at 2000 g for 10 min at 4 degrees and stored in a − 80 freezer. ELISA kits were used to measure estradiol (Calbiotech ES180S-100), progesterone (IBL America, IB791830), and AMH (Anshlabs, AL-113) levels in the serum samples.

### ABT263 gavage

ABT263 gavage was done by following an established protocol^36^. Female C57BL/6J mice at 3 months of age were orally administered either ABT263 (Selleckchem, S1001) at the dosage of 50 mg/kg body weight per day (mg/kg/d) or the vehicle for 7 consecutive days followed by 21 days interval each month over a period of 3 months^36^. The vehicle contained ethanol: polyethylene glycol 400: Phosal 50 PG at the ratio of 10:30:60. Phosal 50 PG is a standardized phosphatidylcholine (PC) concentrate with at least 50% PC and propylene glycol (Phospholipid Gmbh, Cologne, Germany). At the end of the 3-month treatment, blood and ovarian tissues of control mice and ABT-treated mice (now at 6 months of age) were collected for RNA isolation and follicle quantification.

### RNA isolation, RNA sequencing and real-time PCR

Total RNA of each homogenized ovary was extracted using a TRIzol™ Plus RNA purification kit (Invitrogen, 12183555), and each experimental timepoint had three replicates. The RNA samples were submitted to Novogene (Sacramento, California) for mRNA sequencing and analyzed using the NovoMagic Online RNA-seq Bioinformatics Analysis Tool. For real-time PCR experiments, reverse transcription was carried out using 1 μg of total RNA and random primers (Reverse Transcription System, Promega, A3500). IQ™ SYBR® Green qPCR Supermix (Bio-Rad, 1708887) was employed for the real-time PCR assay. PCR reactions were conducted utilizing the Applied Biosystems Real-Time PCR System (Life Technologies, USA). Data normalization was performed using reference genes *B2m* and *Rplp0*. Primer sequences are detailed in Supplementary Table S7.

### Oocyte maturation, DNA damage, and fertility assay

C57BL/6J mice at 9 months of age that were administrated with ABT263 or control vehicles at 3 months of age were injected with 5 IU of eCG (Sigma, G4877). The ovaries were collected at 44 h after the eCG injection. After removing the surrounding fat tissue in PBS, antral follicles were punctured using two insulin needles to release cumulus-oocyte complexes (COCs) containing immature GV-stage oocytes from the ovaries in the in vitro maturation (IVM) medium. The IVM medium was the M2 medium (Sigma, M7167) containing 2 mM glutamine (Sigma, G8540), 3 mg/ml bovine serum albumin (Sigma, A9418), 75 µg/ml penicillin G (Sigma, P3032) and 50 µg/ml streptomycin sulfate (Sigma, S9137). Milrinone (Sigma, 78415-72-2), a selective inhibitor of oocyte-specific phosphodiesterase (PDE3), was added into the IVM medium at the concentration of 5 μM to prevent the GV-stage oocytes from undergoing maturation. Only the oocytes with an intact CV and no apparent signs of degeneration were collected for antibody staining and IVM culture. Milrinone was removed from the IVM medium for oocyte maturation culture^72,73^. The rates of GVBD and PBE were assessed after 2 h and 14 h of IVM culture, respectively. Rate of GVBD = % of GVBD oocytes/total cultured oocytes; rate of PBE = % of PBE oocytes/total cultured oocytes. To assess spindle morphology, oocytes were fixed after 6 h of IVM culture for α-tubulin antibody staining^73^. When collecting oocytes for antibody staining and assessing GVDB, cumulus cells were removed by passing COCs through a hand-drawn fine glass pipette. For oocyte antibody staining, oocytes were fixed in 4% PFA for 1 h and washed in PBST_2_ (PBS with 0.1% Tween-20 and 0.5% Triton X-100) for 1 h, the oocytes were with primary antibodies in PBST_2_ containing 10% donkey serum, 10% BSA and 100 mM Glycine at 4 °C overnight. After washing three times in PBST_2_ for at least 15 min each, the oocytes were incubated with secondary antibodies in PBST_2_ overnight. After washed with PBST_2_, the oocytes were stained with DAPI in PBS to visualize nuclei and imaged using confocal microscopy (Zeiss LSM7000). To measure the width of the metaphase plate (DAPI-positive chromosomes), and the width and length of the meiotic spindle (α-tubulin positive) shown in Fig. 3F, the image of the biggest cross-section from a series of confocal images of each spindle was chosen for the measurement using the Image-J software (ImageJ2, version: 2.14.0/1.54f, http://imagej.net/Contributors). To assess fertility, 1 week after ABT treatment was completed, ten ABT-treated mice and ten control mice at 6 months of age were housed with male C57BL/6J mice at the ratio of one female with one male until 9 months of age, and numbers of the pups of each litter were recorded.

### Statistics

Sample size of the mice used in the present study was determined using power analysis, with a significance threshold of 0.05 and a power level of 80%. Data were analyzed and plotted using Prism 10 (GraphPad Prism 10.1.2). Parametric t-tests were used to examine the difference between two experimental groups. Multiple experimental groups were analyzed using one-way ANOVA with Dunnett’s tests. We assessed the normality of errors and the homogeneity of variances with the Shapiro–Wilk and Bartlett tests, respectively. When the assumptions were not achieved, data were submitted to the logarithmic transformation; however, the results were presented in the original scale. All data was presented as mean ± SD. Differences were considered significant at P < 0.05.

### Supplementary Information

Supplementary Table 2.

Supplementary Table 3.

Supplementary Table 4.

Supplementary Table 5.

Supplementary Table 6.

Supplementary Table 7.

Supplementary Information 7.

## Data Availability

All data generated or analyzed during this study are included in this published article (and its Supplementary Information files). Sequencing data from the RNA-seq experiment are available in the Gene Expression Omnibus (GEO) repository (accession number: GSE253194).
