# Supplementary material for: Primary oocytes with cellular senescence features are involved in ovarian aging in mice
Source: Sci Rep. 2024 Jun 13;14:13606. doi: 10.1038/s41598-024-64441-6 (PMC11176158; doi:10.1038/s41598-024-64441-6)
Supplement: Supplementary file 7 — Supplementary Information 7. [file 41598_2024_64441_MOESM7_ESM.docx]

**Supplementary Information**

**Primary oocytes with cellular senescence features are involved in ovarian aging in mice**

Hao Yan ^1, 6, #^, Edgar Andres Diaz Miranda ^2, #^, Shiying Jin ^2^, Faith Wilson ^2, 3^, Kang An ^2^, Brooke Godbee ^2, 4^, Xiaobin Zheng ^5^, Astrid Roshealy Brau-Rodríguez ^2^, Lei Lei ^2, 3,^ *

1. Buck Institute for Research on Aging, Novato, California, 94945
2. Department of Obstetrics, Gynecology and Women’s Health, University of Missouri School of Medicine, Columbia, Missouri, 65211
3. Division of Biological Sciences, College of Arts and Sciences, University of Missouri, Columbia, Missouri, 65211
4. College of Health Sciences, University of Missouri, Columbia, Missouri, 65211
5. Carnegie Institution for Science, Department of Embryology, Baltimore, Maryland, 21218
6. Current address: Department of Medicine, Stanford University School of Medicine, Stanford, California, 94305

# These authors contributed equally

* Corresponding author

Corresponding author: Lei Lei

Department of Obstetrics, Gynecology and Women’s Health

University of Missouri School of Medicine

**Email:**  lln34@health.missouri.edu

**Supplementary Figure S1.** HMGB1 expression and location in the adult mouse ovary (2 months).

**Supplementary Figure S2.** Primary oocytes with translocated HMGB1 detected by other senescence markers.

**Supplementary Figure S3.** Expression of p16^INK4a^ and p21 in adult mouse ovaries.

**Supplementary Table S1.** Primary oocytes in adult mouse ovaries were negative for apoptosis markers.

**Supplementary Table S2.** Differentially expressed genes between 3-month-old and 6-month-old mouse ovaries.

**Supplementary Table S3.** Differentially expressed genes between 3-month-old and 9-month-old mouse ovaries.

**Supplementary Table S4.** Differentially expressed genes between 6-month-old control and 6-month-old ABT263-treated mouse ovaries.

**Supplementary Table S5.** Differentially expressed genes between 9-month-old control and 9-month-old ABT263-treated mouse ovaries.

**Supplementary Table S6.** Information about antibodies and kits used in the present study.

**Supplementary Table S7.** Information about primers for Realtime PCR used in the present study.

**Supplementary Figure S1**


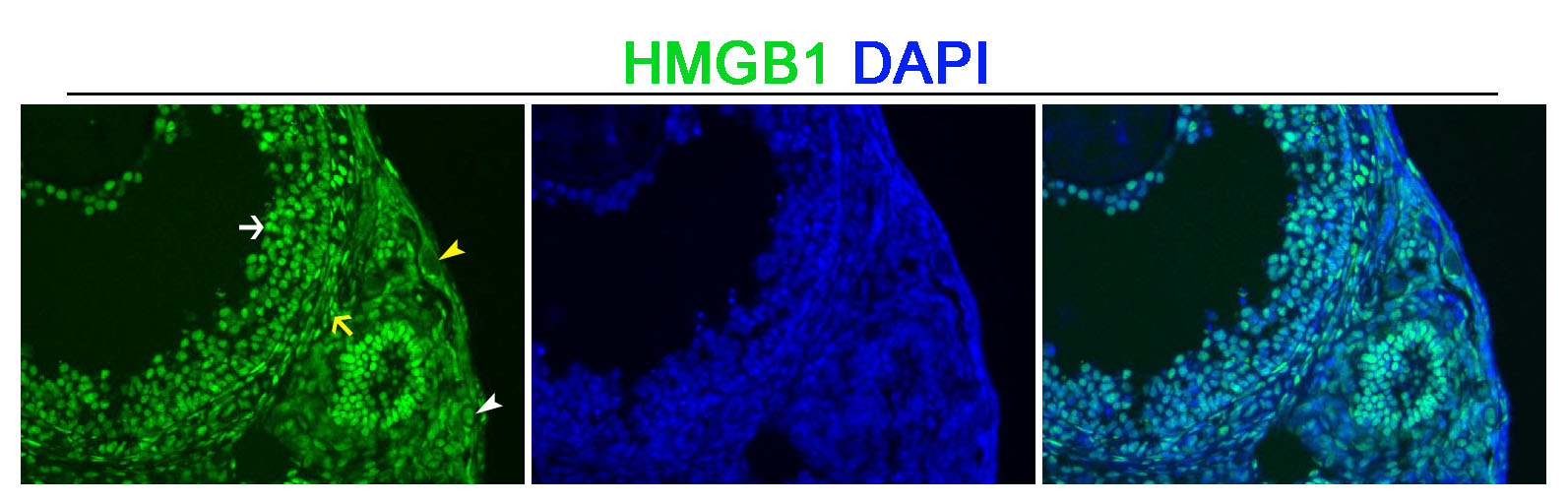


**Supplementary Figure S1.** HMGB1 expression and location in the adult mouse ovary (2 months). HMGB1 was located in the nuclei of granulosa cells (white arrow), interstitial cells (yellow arrow), and the primary oocyte (white arrowhead). Yellow arrowhead showing a primary oocyte with HMGB1 negative staining in the nucleus and cytoplasm.

**Supplementary Figure S2**


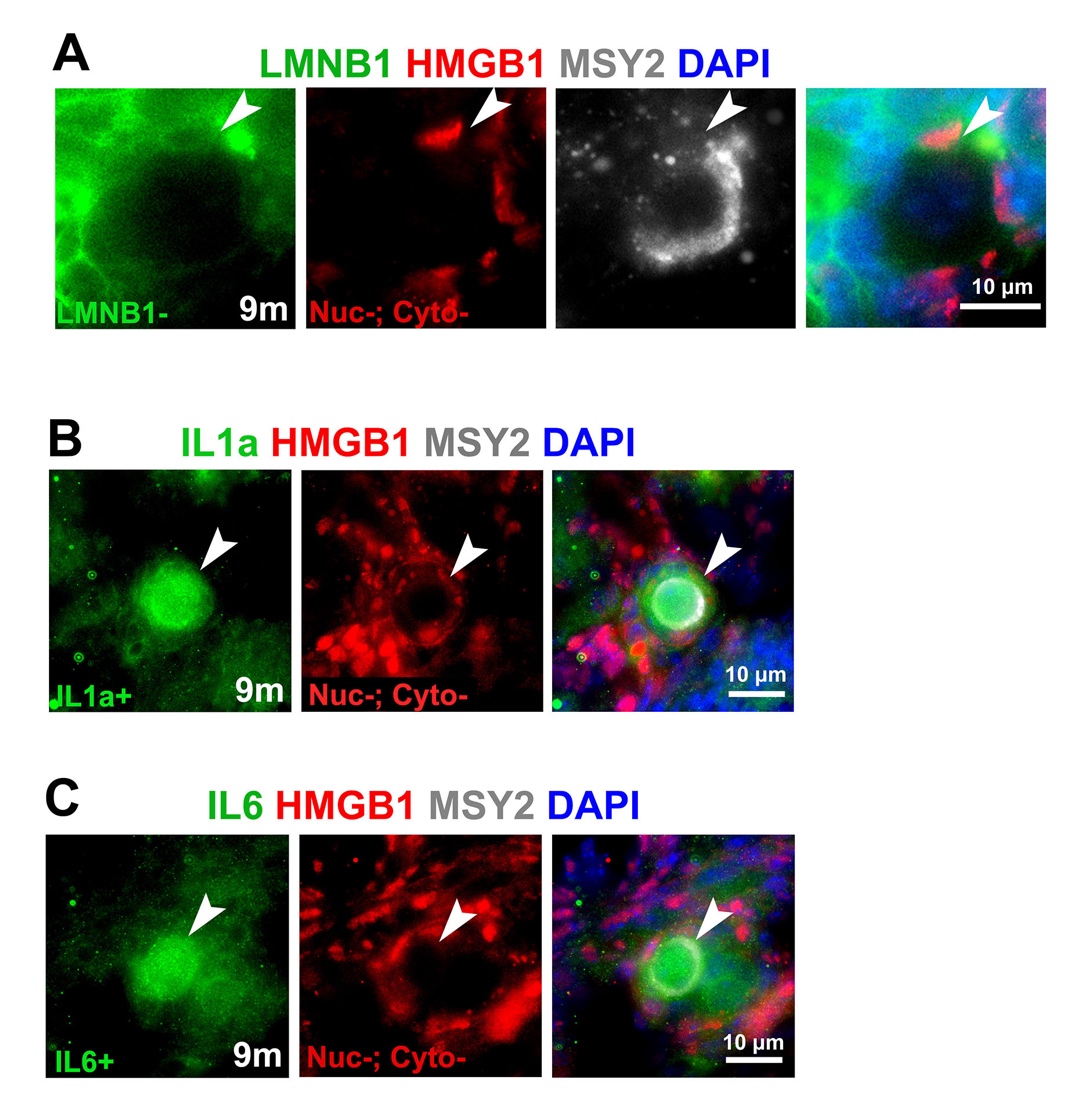


**Supplementary Figure S2.** Primary oocytes with translocated HMGB1 detected by other senescence markers. (A) The primary oocyte had translocated HMGB1 staining (Nuc-; Cyto-) stained negative for lamin b1 (arrowheads, LMNB1-). (B) The primary oocyte had translocated HMGB1 staining (Nuc-; Cyto-) stained positive for IL1a (IL1a+; arrowheads). (C) The primary oocyte had translocated HMGB1 staining (Nuc-; Cyto-) stained positive for IL6 (IL6+; arrowheads).

**Supplementary Figure S3**

**
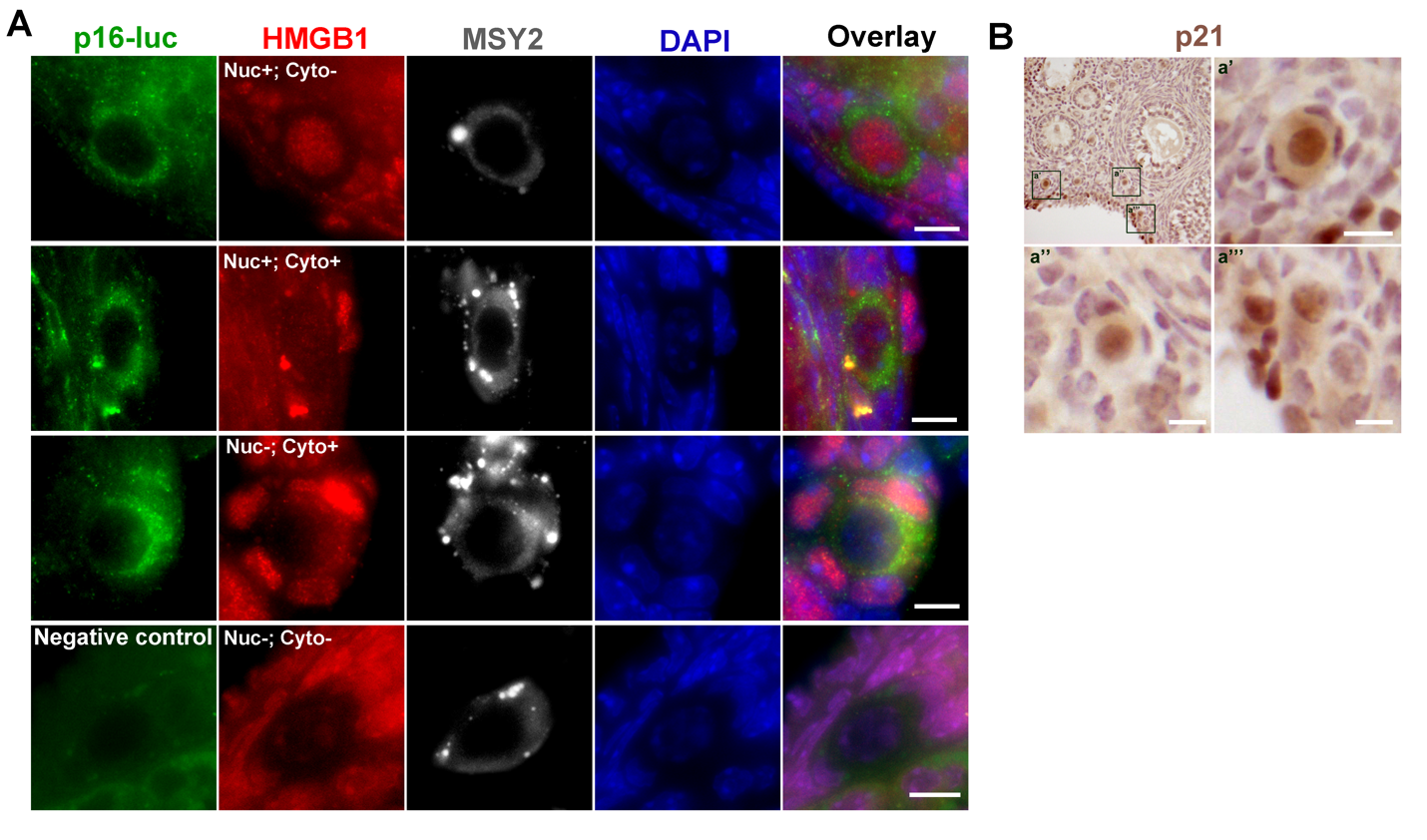
**

**Supplementary Figure S3.** Expression of p16^INK4a^ and p21 in adult mouse ovaries. (A) Antibody staining of luciferase expression driven by p16^INK4a^ in the primary oocytes with nuclear HMGB1 (Nuc+; Cyto-) and translocated HMGB1 in adult (6 months old) p16-3MR mouse ovaries. (B) Antibody staining of p21 expression in adult (6 months old) C57BL/6J mouse ovaries.

**Supplementary Table S1.** Primary oocytes in adult mouse ovaries were negative for apoptosis markers.

|  | 2-month-old ovaries (n=6) | 9-month-old ovaries (n=6) |
| --- | --- | --- |
| Cleaved Caspase-3 positive primary oocytes/ Primary oocytes examined | 0/1282 | 0/1523 |
|  |  |  |
| TUNEL-positive primary oocytes/ Primary oocytes examined | 0/228 | 0/283 |
|  |  |  |
